# AHNAKs roles in physiology and malignant tumors

**DOI:** 10.3389/fonc.2023.1258951

**Published:** 2023-11-14

**Authors:** Shusen Zhang, Zhigang Cai, Hui Li

**Affiliations:** ^1^ Hebei Province Xingtai People’s Hospital Postdoctoral Workstation, Xingtai, China; ^2^ Postdoctoral Mobile Station, Hebei Medical University, Shijiazhuang, China; ^3^ Department of Pulmonary and Critical Care Medicine, Affiliated Xing Tai People Hospital of Hebei Medical University, Xingtai, China; ^4^ The First Department of Pulmonary and Critical Care Medicine, The Second Hospital of Hebei Medical University, Shijiazhuang, China; ^5^ Department of surgery, Affiliated Xing Tai People Hospital of Hebei Medical University, Xingtai, China

**Keywords:** AHNAK, AHNAK2, biological function, malignant tumors, prognosis

## Abstract

The AHNAK family currently consists of two members, namely AHNAK and AHNAK2, both of which have a molecular weight exceeding 600 kDa. Homologous sequences account for approximately 90% of their composition, indicating a certain degree of similarity in terms of molecular structure and biological functions. AHNAK family members are involved in the regulation of various biological functions, such as calcium channel modulation and membrane repair. Furthermore, with advancements in biological and bioinformatics technologies, research on the relationship between the AHNAK family and tumors has rapidly increased in recent years, and its regulatory role in tumor progression has gradually been discovered. This article briefly describes the physiological functions of the AHNAK family, and reviews and analyzes the expression and molecular regulatory mechanisms of the AHNAK family in malignant tumors using Pubmed and TCGA databases. In summary, AHNAK participates in various physiological and pathological processes in the human body. In multiple types of cancers, abnormal expression of AHNAK and AHNAK2 is associated with prognosis, and they play a key regulatory role in tumor progression by activating signaling pathways such as ERK, MAPK, Wnt, and MEK, as well as promoting epithelial-mesenchymal transition.

## 1 Introduction

The AHNAK family currently contains two members, AHNAK (AHNAK1) and AHNAK2, which share approximately 90% homologous sequences with some similarities in molecular structure and biological function (1).

AHNAK, or AHNAK Nucleoprotein, is also known as Desmoyokin. In 1989, Hieda et al. isolated and identified a new desmosomal plaque protein, Desmoyokin, at the periphery of the desmoplasmic plaques in the bovine oral stratified squamous epithelium (2). In 1992, Shtivelman et al. found a giant protein of round about 700 kDa in size when screening for genes that might be reduced or absent in neuroblastoma cells, and then named it AHNAK (which means giant in Hebrew) (3). Subsequent studies by Hashimoto et al. confirmed that AHNAK and Desmoyokin refer to the same protein (4). AHNAK was initially thought to be encoded by a 17-kb intronless gene on human chromosome 11q12 (5). However, it has been recently reported that the gene contains a giant exon flanked by introns and multiple small exons encoding a giant protein (~700 kDa) and a small protein (17 kDa), and that the commonly studied AHNAK is classified as a giant protein of 700 kDa (6). AHNAK consists of three structural domains: an amino terminal containing 251 amino acids and PDZ domain (N-terminal), a central repeating units (CRUs) containing multiple repeating units of 4,392 amino acids (about 128 residues per repeating unit), and a carboxyl terminal (C-terminal) containing 1002 amino acids, where there are more interaction and regulatory sites (7). AHNAK can be localized in the nucleus, cytoplasm, cell membrane, lysosomes, mitochondria and other structures. Its localization is variable, and the changes may be involved in regulating different functions. As report goes, the cellular localization of AHNAK is regulated by the C-terminal (8).

AHNAK2, or AHNAK Nucleoprotein 2, is also known as C14orf78. It is the second member of the AHNAK family and was originally found in mouse heart extracts (9). AHNAK2 is a giant protein with a molecular weight of over 600 kDa with its coding gene located on human chromosome 14q32, possessing an open reading frame of 15 kb (9). Slightly different from AHNAK, AHNKA2 has at least seven exons, six of which are relatively small together with a large exon of approximately 17,559 bp (9). AHNAK2 contains a total of 5,795 amino acids and consists of a three-part structure: a short non-repetitive N-terminal containing the PDZ structural domain, a central structural domain consisting of 24 repeat units (each containing 165 amino acids) forming the central structural domain, and a C-terminus with a molecular weight size of approximately 100 kDa (9, 10).

This review will focus on the biological functions of AHNAK family members with an emphasis on their role in the development of malignant tumors.

## 2 Functional introduction to the AHNAK family

**Figure 1:**
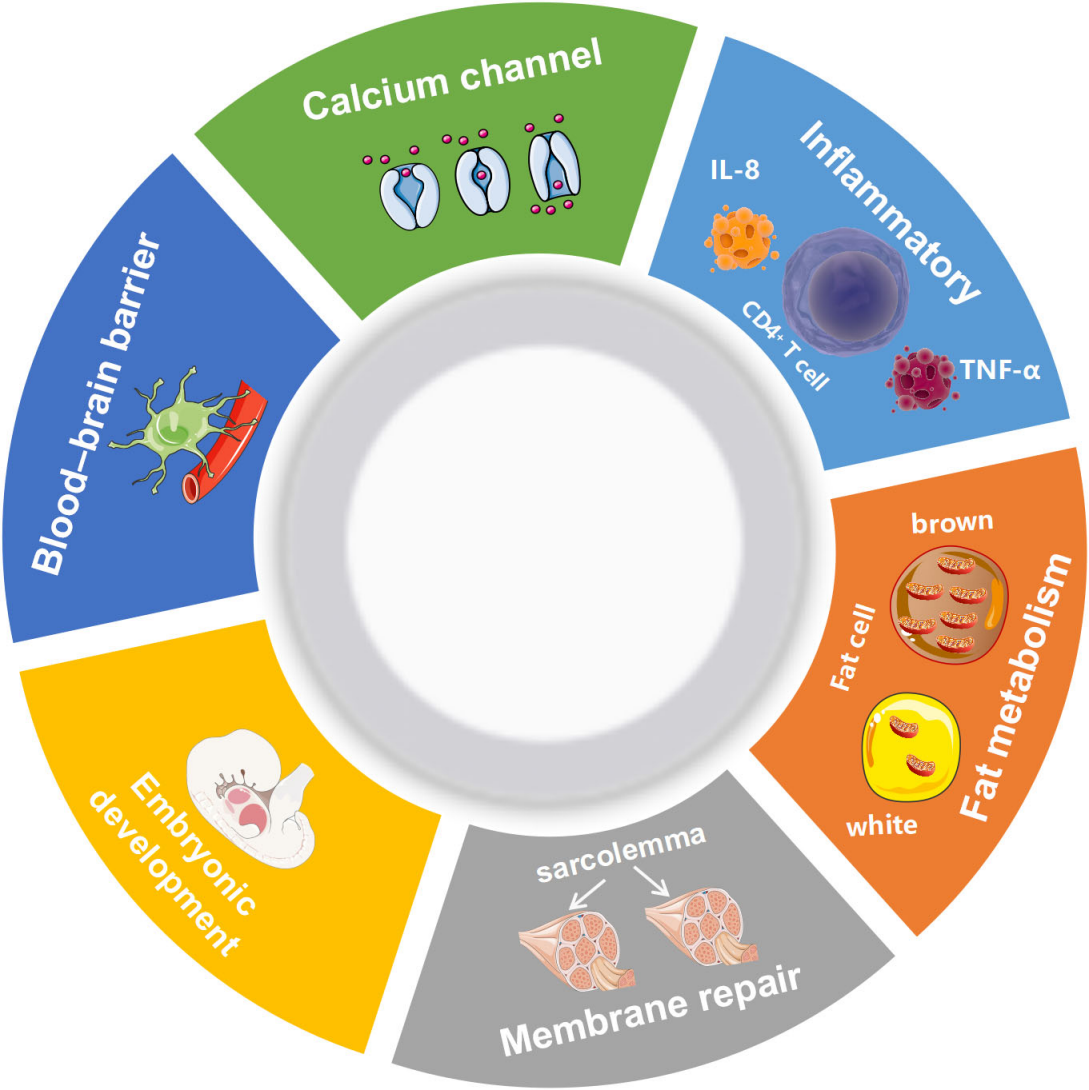
Biological processes regulated by AHNAK. TNF-α, Tumor necrosis factor; IL-8, Interleukin 8.

**Figure 2:**
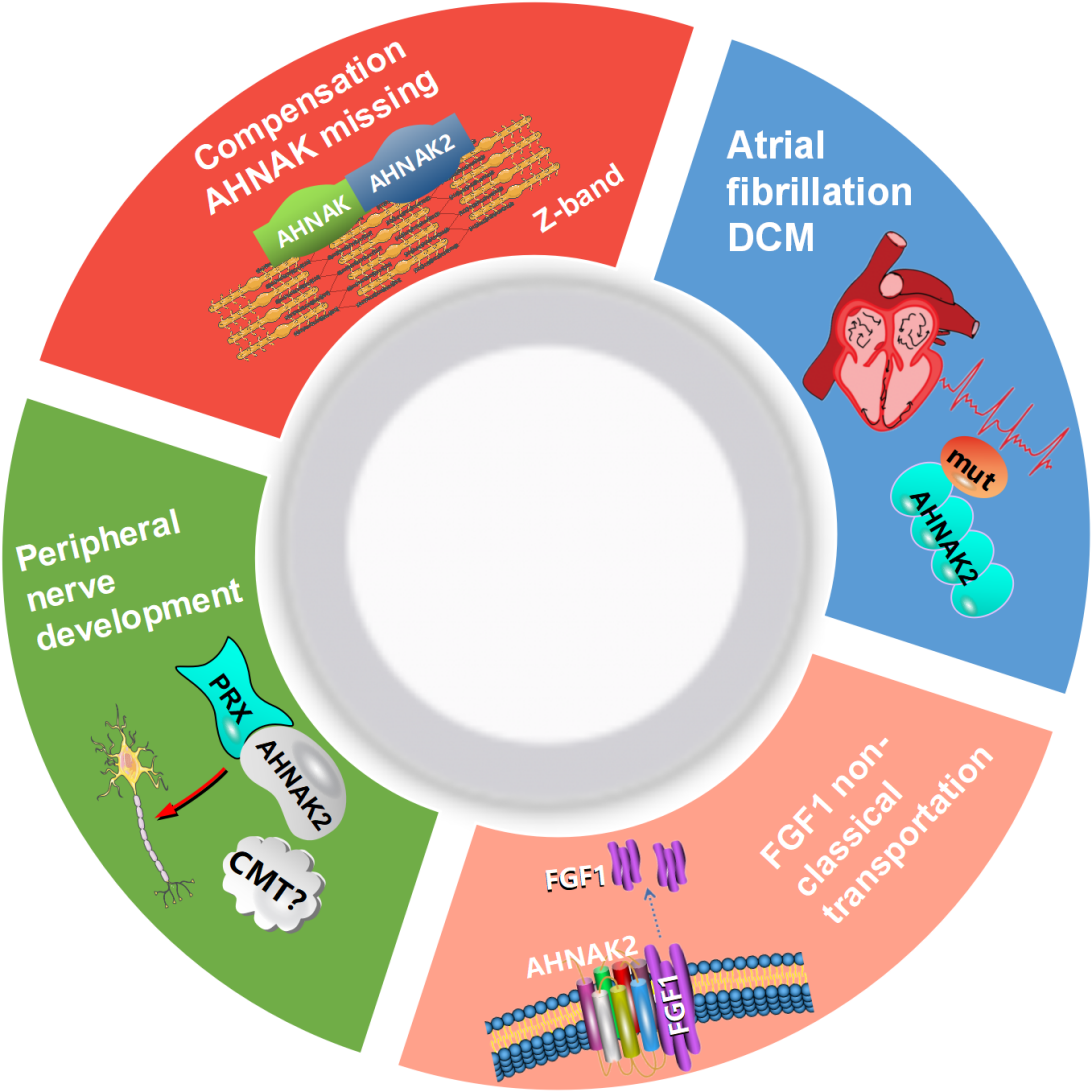
Biological processes regulated by AHNAK2. CMT, Charcot-Marie-Tooth; DCM, dilated cardiomyopathy; PRX, periaxin; FGF1, fibroblast growth factor.

AHNAK can be involved in a wide range of biological processes (**Figure 1**). It has been recently reported that AHNAK plays a critical role in the regulation of calcium channels, blood-brain barrier formation, embryonic development, lipid metabolism, membrane repair, inflammatory responses and other processes (11). However, compared to AHNAK, the function of AHNAK2 is still relatively poorly studied and its biological function is not well defined. AHNAK2 may play a significant part in sarcolemma assembly, peripheral nervous system development, and cardiac calcium channel regulation (**Figure 2**). In the following sections, we will briefly review the biological function of AHNAK family.

### 2.1 Calcium channel regulation

Several studies have confirmed that AHNAK plays an important role in the regulation of calcium channel, in particular L-type voltage gated calcium channels (LVGCC) (1, 12). In cardiomyocytes, AHNAK co-localizes with the β2 subunit of cardiomyocyte L-type calcium channels (Cavβ2) in the myocardium and inhibits LGVCC activity by binding to Cavβ2 through the C-terminal, which will be released for normal function and increased inward calcium flux upon β-adrenergic activation and subsequent protein kinase A (PKA) phosphorylation, thereby regulating cardiomyocyte contraction (13, 14). AHNAK2 and AHNAK can be localized together in the sarcolemmas and Z-bands of mouse cardiomyocytes. It showed no obvious abnormalities to knockdown the AHNAK in mouse models, which suggested that AHNAK2 may compensate for the absence of AHNAK (9). ANRIL is an lncRNA gene whose transcript target is down-regulated in patients with Coronary Artery Disease (CAD) and is involved in the initiation process of CAD. In contrast, decreased expression of AHNAK2 was detected after ANRIL knockdown (15). In patients with atrial fibrillation (AF) and dilated cardiomyopathy, AHNAK2 was one of the three most frequently mutated genes, with respective mutation rates of 52% and 51% (16, 17). These results suggest that AHNAK2 may participate in the regulation of heart disease, although the specific molecular mechanism is not clear. AHNAK is an important player in the regulation of cardiac L-type calcium channels. As its homologue, does AHNAK2 influence the development of cardiac disease by regulating L-type calcium channels? Further studies are needed to clarify its role.

In skeletal muscle, AHNAK can provide mechanical stability during muscle contraction and co-localizes with the β1 subunit of skeletal LVGCC at the T-tubule to regulate skeletal muscle contraction in a manner similar to Cavβ2 regulation (18). In neurons, AHNAK can participate in the regulation of Cav1.2 and Cav1.3 channels, enhancing neuronal excitability and increasing excitatory neurotransmitter release. Besides, in inhibitory synapses, AHNAK can interact with Cavβ4 and participate in its regulation (in a manner similar to Cavβ2 regulation), reducing inhibitory neurotransmitter release and thus ameliorating depressive behavior (19, 20). Additionally, in the presence of arachidonic acid, the center recall unit (CRU) region of AHNAK activates phospholipase C and produces inositol triphosphate and diacylglycerol, which in turn regulate intracellular calcium flux (21). The complete actin cytoskeleton plays a critical part in the regulation of calcium channel currents. AHNAK can interact with G-actin and F-actin, and can participate in cytoskeletal regulation, consequently affecting calcium channel function (22).

### 2.2 Blood-brain barrier formation

The blood-brain barrier forms the basis for brain homeostasis and low permeability (23). It has been shown that AHNAK is expressed on the plasma membrane of endothelial cells that form the blood-brain barrier but is not found in capillary endothelial cells that undergo molecular exchange between blood and extracellular fluid. Co-culture of astrocytes with brain capillary endothelial cells resulted in the relocalization of AHNAK from the cytoplasm to the plasma membrane when endothelial cells acquired blood-brain barrier properties (24). In the case of spinal cord injury, increased expression of AHNAK was detected in cells with barrier properties and might be involved in the formation of a barrier around the injury parts (25).

### 2.3 Embryonic development

M. Downs et al. detected AHNAK expression in mouse trophoblast ectoderm derivatives, in the urinary bladder attached to the chorion, and at the tip of the neural plate during neural tube formation (26). A study proved that AHNAK is involved in the formation of germinal bodies and the initial differentiation of pluripotent stem cells by inhibiting c-Myc (27). Impaired migration of enteric neural crest cells during embryonic development leads to a lack of ganglion cells in the distal part of the digestive tract, resulting in congenital megacolonization. It has been reported in studies that there is significant upregulation of AHNAK protein expression and inhibition of cell migration and proliferation in stenotic intestinal tissues from patients with congenital megacolon (28).

### 2.4 Muscle membrane repair

Dysferlin is a membrane protein involved in skeletal muscle membrane repair, with which AHNAK can co-localize and interact to participate in the sarcolemma repair process (29). During this process, S100A10, annexinA2 and AHNAK can bind to form a complex that promotes faster recruitment of AHNAK proteins to the plasma membrane, then inducing more efficient membrane repair (30).

### 2.5 Lipid metabolism

Studies show that AHNAK deficiency in mice on a high-fat diet leads to increased insulin sensitivity and that AHNAK plays an important role in β-adrenergic signaling to regulate white adipose tissue browning, catabolism and thermogenesis (31). In hepatocytes, AHNAK deficiency induced upregulation of fibroblast growth factor 21 (FGF21) expression and attenuated fatty liver formation in mice fed a high-fat diet (32). Jong et al. put forward that AHNAK can participate in the regulation of adipocyte differentiation via members of the bone morphogenetic protein family, such as human hone morphogenetic protein 2 (BMP2) and human hone morphogenetic protein 4 (BMP4) (33, 34). These findings confirm that AHNAK plays a critical part in fat metabolism and may have implications for the treatment of obesity or related metabolic diseases.

### 2.6 Inflammatory response

Studies have found that AHNAK (5758-5775 polypeptide segment) can activate mast cells to release IL-8 and TNF-α, mediated by suppression of tumorigenicity 2 (ST2), and participate in the development and progression of psoriasis (35). In the single nucleated cells and membrane tissues peripheral blood from patients with habitual abortion, the mRNA and protein levels of AHNAK are elevated. And AHNAK with increased CD4+ T cell expression may be involved in the immune dysregulation of RPL in habitual abortion by elevating IL-6 production (36).

### 2.7 Other functions

In peripheral nerves, AHNAK is of much importance in the formation and maintenance of myelin sheaths (37). Periaxin (PRX) is a protein abundant in the peripheral nervous system and has an important role in the cytoskeleton and myelin formation (38). Both PRX and AHNAK2 have PDZ structures and share more than 50% of the homologous sequences in this region. One report points out that AHNAK2 and PRX can form homodimers, suggesting that AHNAK2 may be involved in regulating cytoskeleton and peripheral nerve development, although further research are required to confirm (39). Charcot-Marie-Tooth (CMT) disease is an inherited peripheral neuropathy affecting motor and sensory neurons. A study using linkage analysis and whole exome sequencing revealed significantly lower mRNA and protein levels of AHNAK2 in fibroblasts from patients with AR-CMT (autosomal recessive CMT), suggesting that AHNAK2 may be involved in the pathogenesis of autosomal recessive CMT in Malaysia (40). These results suggest that the AHNAK family may be involved in the regulation of peripheral nervous system development.

According to research findings, AHNAK/Annexin A2 complex influences the cortical organization of the astral microtubule anchoring complex, and thereby mitotic spindle positioning in human cells (41). One report showed that AHNAK knockout mice had shortened femur and tibia with significantly decreased skeletal strength, as well as morphological abnormalities in the coccyx with age (42). In addition, as a substrate of calpain-3, AHNAK may be closely associated with calpain-specific limb-girdle muscular dystrophy 2A (43). It is suggested that AHNAK may play an important role in skeletons and skeletal muscles development and metabolism. Cleavage of autoantigens by granzyme B and caspase 3 is commonly seen in systemic autoimmune diseases. AHNAK can be cleaved by the both enzymes and identified as a systemic lupus erythematosus (SLE) autoantigen, promising a new target for therapy (44). Although further studies are needed to confirm, AHNAK2 has been identified as a susceptibility gene for SLE by exon sequencing of large samples (45). It has been reported that AHNAK2 and fibroblast growth factor 1 (FGF1) are co-localized near the cell membrane, which are important components in the regulation of non-classical transport of FGF1. It is a non-classically released growth factor that plays an important role in regulating cell growth, tumor invasion, angiogenesis and participates in the regulation of MAPK-ERK and other signaling pathways (46). The role of AHNAKs in malignant tumors has received much attention and will be discussed in detail below.

## 3 Relationship between AHNAK family and tumor development

### 3.1 Lung cancer

#### 3.1.1 Lung adenocarcinoma

A study on Lung adenocarcinoma found that UBE3C enhances A549 cell stemness by ubiquitinating the degradation of AHNAK, which in turn disrupts the AHNAK-P53 complex, and that patients with high AHNAK expression have longer survival (47). Another study seemed to confirm the above results, stating that AHNAK expression was decreased in human lung adenocarcinoma, that AHANK-/- mice showed increased lung volume, alveolar wall thickening, and type II alveolar epithelial cell proliferation, and that approximately 20% of aged AHNAK-/- mice developed lung tumors and were more susceptible to lung cancer under urethane induction (48). The above studies seem to indicate a possible oncogenic role of AHNAK in NSCLC. However, it has also been reported that AHNAK expression was elevated during TGF-β induced epithelial mesenchymal transition in human lung adenocarcinoma A549 cells, suggesting that AHNAK may promote invasion and metastasis of Lung adenocarcinoma cells (49).

While the role of AHNAK in lung adenocarcinoma remains controversial, several studies have now confirmed the role of AHNAK2 as a pro-cancer factor in lung cancer. It was reported that AHNAK2 expression was upregulated in lung adenocarcinoma and correlated with poor prognosis, which may be an independent factor in determining prognosis. In addition, the migration of lung adenocarcinoma A549 cells was inhibited by AHNAK2 knockdown (50). Liu et al. reported that downregulation of AHNAK2 inhibited TGF-β1-induced cell migration, invasion, and EMT, and decreased Smad3 signaling activation. Inclusion of Smad3 phosphorylation inhibitors in lung adenocarcinoma cells did not affect the regulation of cell migration, invasion and EMT by TGF-β1 with or without knockdown of AHNAK2, suggesting that AHNAK2 promotes lung adenocarcinoma progression through the TGF-β1/Smad3 pathway (51). It was also reported that downregulation of AHNAK2 expression inhibited the phosphorylation of ERK, which inactivated the MAPK signaling pathway and led to the proliferation and migration of lung adenocarcinoma cells (52). A study showed that AHNAK2 expression in lung adenocarcinoma was negatively correlated with activated B cells, activated CD8+ T cells and immature B cell infiltration, while it was positively correlated with central memory CD4+ T cells, tumor-associated macrophages, M1 macrophages and M2 macrophage infiltration, promising a new target for immunotherapy (53).

#### 3.1.2 Lung squamous carcinoma

Radiation sensitivity index (RSI) may predict the sensitivity of radiotherapy. A study collected and analyzed lung squamous cancer cell carcinoma datasets from both TCGA and GEO (GSE73403 and GSE37745) databases and found significant differences in AHNAK2 expression between high and low RSI groups, suggesting that AHNAK2 may be related to the molecular mechanisms of regulating radiotherapy sensitivity in lung squamous carcinoma (54).

#### 3.1.3 Mutation of AHNAK2 in non-small cell lung cancer

A study reported that The Cancer Genome Atlas (TCGA) database revealed a high mutation rate of AHNAK2 in lung cancer, which reached 18.8%. And a collection of 12 specimens from patients with brain metastases from lung cancer was sequenced, and the mutation rate of AHNAK2 in lung cancer patients with brain metastases was found to be as high as 26.9% (55). Cui et al. found that approximately 11% of NSCLC patients carry AHNAK2 mutations and defined those with a score <-2.5 as deleterious mutations by the PROVEAN tool. Del-AHNAK2-mut was further found to be strongly associated with tumor mutational burden (TMB), neoantigen load (NAL) levels and tumor-infiltrating immune cell (TIIC) to better predict patient PFS and OS (56). These results suggested that mutations in AHNAK2 may potentially be involved in the regulation of non-small cell lung cancer progression.

### 3.2 Melanoma

HACAT cells, human immortalized keratin-forming cells, were derived from normal skin at the periphery of the lesion of a 62-year-old male melanoma patient, which showed reduced migratory and invasive capacity after AHNAK knockdown. Treatment of the highly metastatic and tumorigenic melanoma cell line B16F10 with TGF-β and shAHNAK further confirmed that AHNAK knockdown resulted in a significant reduction in N-calmodulin expression and Smad3 phosphorylation and that TGF-β did not induce metastasis and invasion in AHNAK knockdown B16F10 cells (57). A study by Suh et al. also confirmed that AHNAK -/- mice were more resistant to lung metastasis by B16F10 cells than wild type mice (58). However, two other studies presented opposite findings. Huang et al. reported that AHNAK expression was downregulated in primary malignant melanoma PMM in ureteral tissue compared to paracancerous ureteral tissue (59). Another study noted that AHNAK expression was significantly downregulated in melanoma and correlated with poor prognosis, and that knockdown of AHNAK in primary melanocytes resulted in reduced expression of E-calcineurin (60). The role of AHNAK in melanoma development still appears to be controversial and needs to be further investigated.

Analysis of the data provided by TCGA and Hodis et al. showed that mutations in AHNAK2 were more common in melanoma patients of older age (over 40 years old). However, the significance of this phenomenon is unclear and has not been further explained or investigated by the authors (61). Li et al. reported that AHNAK2 was highly expressed in uveal melanoma (UM), which is closely associated with high expression and shorter survival in UM, and that the expression of AHNAK2 was higher in primary tumor tissues with metastatic UM than that in primary tumor tissues without metastatic UM. Compared to corneal epithelial cell line D78, the expression of AHNAK2 was significantly upregulated in UM cell lines M17 and SP6.5. And knockdown of AHNAK2 in UM cell lines inhibited the activation of PI3K signaling and reduced cell proliferation, migration and invasion (62).

### 3.3 Glioma

Zhao et al. examined the expression of AHNAK in six glioma cell lines. Compared to normal glial cell lines (HEB), AHNAK mRNA levels were downregulated in four cell types (SHG-44, A172, U87, U251), especially in the A172, U87 and U251 cell lines (p<0.001). In addition, 30 normal brain tissue and 73 glioma tissue specimens were collected in this study, which confirmed that the low expression of AHNAK in glioma was correlated with poor prognosis (63). Overexpression of AHNAK in U87 and U251 cells inhibited the proliferation and invasion of glioma cells and induced apoptosis (63). Another study using TCGA database analysis showed that AHNAK transcript levels were significantly reduced in glioma stem cells with greater proliferation and migration capacity compared to differentiated glioma cells, although there was no significant difference of AHNAK expression in glioblastoma (GBM) and normal brain tissue (64). These results seem to indicate that AHNAK plays an inhibition role in gliomas. Moreover, the prognosis of glioblastoma patients with AHNAK mutations is worse (median survival of 4.16 months for AHNAK mutated patients compared to 13.53 months for wild-type AHNAK patients), which has been confirmed to act as an independent factor for poor prognosis in GBM (64).

Research in the relationship between AHNAK2 and glioma is still lacking. The Glioma Centre of Southern Medical University reported a rare case of isocitrate dehydrogenase (IDH) wild-type epithelioid glioblastoma with a BRAF V600E mutation and good results with virofenib treatment, in which the AHNAK2 mutation was identified in the whole exome sequencing results, promising a new target for the treatment of GBM (65).

### 3.4 Laryngeal cancer

A study showed that in normal tissues AHNAK showed only weak or moderate staining with no strong staining present, whereas in laryngeal cancer tissue specimens AHNAK strong staining accounted for 25% (21/83), in which tumor tissue specimens were collected from 83 laryngeal cancer patients and 12 normal tissue specimens (9 tonsils and 3 healthy epithelial tissues of the palatal suspensor) for immunohistochemical analysis. And the patients with high AHNAK expression had a worse prognosis (66). In another study, hanfungin was found to inhibit the growth of laryngeal cancer Hep-2 cells by upregulating AHNAK expression (67). The role of AHNAK in the two reports appears to be inconsistent and needs further investigation. Studies on the relationship between AHNAK2 and laryngeal cancer are lacking. The role of AHNAK2 is not clear.

### 3.5 Thyroid cancer

In a study of papillary thyroid carcinoma (PTC) in China, tumor and normal tissue specimens from 16 PTC patients were collected and high-throughput sequencing was performed. The analysis showed that AHNAK may be a driver gene for the development of PTC (68).

Compared with AHNAK, AHNAK2 in thyroid cancer has been relatively well studied and been considered to influence thyroid cancer progression as a pro-carcinogenic factor. Several studies using bioinformatics analysis have shown that AHNAK2 expression is upregulated in PTC, indicating poor prognosis. Additional functional analysis has shown that AHNAK2 is closely associated with immune cell infiltration and may contribute to thyroid cancer progression by regulating cell adhesion, cell junctions and immune-related pathways (69, 70). Ye et al. confirmed through various experiments that AHNAK2 is upregulated in thyroid cancer tissues, especially in metastatic thyroid cancer, where its expression is higher, and that high expression of AHNAK2 suggests poor prognosis (71). It was also found that AHNAK2 may achieve regulation of thyroid cancer migration, invasion and lymph node metastasis through the NF-κB signaling pathway (71).

### 3.6 Breast cancer

MMTV-PyVT (Mouse mammary tumor virus, Polyoma Virus middle T antigen) mice carry murine mammary tumor virus and females develop significant mammary tumors as early as 5 weeks, which are often used in human breast cancer research. The study successfully screened MMTV mice with AHNAK-/- and AHNAK+/+ phenotypes and found that AHNAK-/- mice developed more mammary tumors. It was also found that AHNAK expression was decreased in breast cancer tissues compared to normal tissues, and it was hypothesized that AHNAK may be a tumor suppressor and that its deficiency promoted mammary cell proliferation and tumor development in mice (72). Using the TCGA and METABRIC databases, J. Cimas et al. found that AHNAK is mutated in approximately 5% of basal cell-like breast cancers and that AHNAK mutations are associated with a good prognosis (73). Studies on AHNAK2 and breast cancer are lacking. The biological role of AHNAK2 needs to be further investigated.

#### 3.6.1 Triple-negative breast cancer

The production of vesicles by cancer cells, which promote the migration of recipient fibroblasts, plays an important part in promoting fibroblast migration and regulating the tumor microenvironment. One report showed that AHNAK is more highly expressed in vesicles produced by MDA-MB-231 cells from highly invasive breast cancers compared to the less invasive MC7 cells, and appears to be an essential element for vesicle formation. Treatment of non-transformed fibroblasts with vesicles from MDA-MB-231 cells improved their migratory capacity. The study also found that AHNAK expression was upregulated in invasive ductal carcinoma and metastatic carcinoma compared to normal breast epithelium, with higher expression in metastatic carcinoma in particular (74). These data seem to suggest that AHNAK is abnormally highly expressed in breast cancer and that it can promote tumor progression by inducing fibroblast migration and extracellular matrix destruction around Triple-negative breast cancer (TNBC) cells. However, some studies have suggested the opposite that AHNAK is a suppressor in breast cancer. Chen et al. reported that AHNAK mRNA levels were significantly downregulated in human breast cancer cell lines, particularly in triple-negative breast cancer cell lines, and that downregulation of AHNAK expression was associated with poor prognosis in TNBC. *In vivo* and *in vitro* experiments confirmed that AHNAK can regulate signaling pathways such as AKT/MAPK and Wnt/βcatenin to inhibit proliferation and metastasis of TNBC cells (75). In doxorubicin-resistant triple-negative breast cancer cells, AHNAK knockdown was found to prevent a decrease in doxorubicin-regulated activated caspase 7 expression as well as an increase in S-phase, whereas AHNAK overexpression decreased activated caspase 7 expression and induced an increase in S-phase, suggesting that AHNAK may be involved in the regulation of chemoresistance in TNBC (76). The roles of AHNAK and AHNAK2 in lung and breast cancer subtypes are compared in **Table S1**.

### 3.7 Hepatocellular carcinoma

A study examined AHNAK mRNA levels in 60 liver cancer tissues and paracancerous tissues and found that AHNAK expression was significantly elevated in liver cancer tissues, in which the methylation level of AHNAK promoter in peripheral blood mononuclear cells (PBMC) from 260 patients with varying degrees of liver disease was also measured (77). Interestingly, the level of AHNAK methylation was negatively correlated with disease severity, with 44.44% methylation in the normal control group, 38.23% in the chronic hepatitis B group, 34.38% in the compensated cirrhosis group, 31.43% in the decompensated cirrhosis group, and the lowest in the liver cancer group, about 27.7%. It was demonstrated by receiver operating characteristic (ROC) curves that AHNAK methylation can serve as a marker for the diagnosis of hepatocellular carcinoma (Area Under Curve, AUC=0.98). Methylation of CpG islands in the promoter region is known to cause gene silencing, resulting in reduced or absent gene expression. In this study, AHNAK showed hypomethylation and increased expression in HCC, suggesting that AHNAK may act as a cancer promoter. Another study found that GATA binding protein 4 (GATA4) was highly expressed in hepatoblastoma and correlated with the mesenchymal migration phenotype of hepatoblastoma cells, and that GATA4 gene silencing inhibited the migration of HUH6 cells (78). While AHNAK expression decreased after GATA4 knockdown, overexpression of GATA4 caused AHNAK elevation, suggesting that AHNAK upregulation may promote hepatoblastoma progression. Li et al. found that AHNAK can co-localize and interact with insulin-like growth factor 1 (IGF-1R) and promote the growth of hepatocellular carcinoma (HCC) (79). Contrary to the findings reported above, Rui et al. found that upregulated ring finger protein 38 (RNF38) promoted TGF-β signaling through ubiquitinated degradation of AHNAK and induced epithelial-mesenchymal transition in HCC cells. Furthermore, AHNAK knockdown restored the reduced migratory and invasive capacity of HCC cells due to RNF38 downregulation, which appears to indicate the presence of AHNAK as an oncogenic factor in hepatocellular carcinoma (80).

AHNAK2 has rarely been studied in HCC. A study collected 39 specimens of HCC patients from China, 22 of which were subjected to whole exome sequencing, and revealed a high mutation rate in AHNAK2 (22.7%, 5/22), although the high mutation rate in AHNAK2 was not explored in more detail (81).

### 3.8 Pancreatic cancer

Studies have confirmed that AHNAK is aberrantly highly expressed in pancreatic ductal adenocarcinoma (PDAC) and is associated with shorter disease-free survival and poorer overall survival. Knockdown of AHNAK induced P53 downregulation and inhibited EMT, proliferation and migration of PDAC cells, while addition of P53 protein reversed the effect of AHNAK downregulation on PDAC cells, which suggests that AHNAK may be a novel biomarker for PDAC (82).

Bioinformatic analysis has shown that AHNAK2 expression is upregulated in PDAC. Several studies have demonstrated high diagnostic and predictive prognostic value of clinical prediction models for PDAC constructed with AHNAK2 as a factor (83–85). Specific molecular mechanism studies of AHNAK2 in PDAC are still lacking and need to be further explored and verified.

### 3.9 Esophageal cancer

One study indicated that SMYD2 can directly methylate AHNAK as well as multiple sites in the CRU region of AHNAK2, which may be involved in regulating cell adhesion, cell signaling, and tumor cell migration and invasion (86). Hou et al. reported that AHNAK2 may be involved in the regulation of radioresistance in esophageal cancer. Knockdown of AHNAK2 resulted in increased radioresistance in esophageal cancer KYSE-150 cells. Whole-exome sequencing analysis of esophageal cancer cells with AHNAK2 knockdown revealed that AHNAK2 may act through NF-κB and TNF signaling pathways to regulate the expression of interleukins, interleukin receptors and chemokines (87).

### 3.10 Gastric cancer

Studies have shown that upregulation of MicroRNA-93-5p suppressed AHNAK expression, which activated the Wnt signaling pathway and promoted EMT, proliferation, and migration of gastric cancer cells. In contrast, overexpression of AHNAK inhibited the migration, invasion and EMT of gastric cancer HGC-27 cells, which indicates that AHNAK may act as a suppressor to regulate the progression of gastric cancer (88, 89).

Epstein-Barr virus (EBV)-associated gastric cancer (GC) is characterized by high DNA methylation and is more sensitive to 5-fluorouracil and cisplatin chemotherapy. It was reported that AHNAK2 methylation was increased in EBVGC cells compared to normal GC cells. And decreased expression of AHNAK2 in EBVGC metastatic sites was confirmed using IHC. It has also been reported that AHNAK2 may be involved in the regulation of 5-fluorouracil and cisplatin resistance. In summary, Ohmura et al. speculated that gene silencing due to increased AHNAK2 methylation may mediate the regulation of EBVGC sensitivity to chemotherapy, although detailed molecular mechanistic studies are still required to confirm their speculation (90). One study found that gastric cancer patients with PIK3CA, LRP1B and AHNAK2 mutations had a better prognosis, and that investigating the molecular mechanisms of the three-gene interaction has potential value in improving the prognosis of gastric cancer (91). Another study constructed a clinical prediction model for gastric cancer using AHNAK2 as a factor and validated that the model has some predictive value for recurrence and prognosis (92).

### 3.11 Colorectal cancer

In colorectal cancer, SORBS1 could co-localize with AHNAK and induce ERK phosphorylation and up-regulation of ROCK1 expression through inhibition of AHNAK expression, which promoted the proliferation and migration of colorectal cancer cells, suggesting that AHNAK may act as a suppressor in colorectal cancer (93).

AHNAK2 was identified as a differentially expressed protein in colorectal cancer RKO cells possessing KRAS mutations, without Ras pathway mutations. In addition, AHNAK2 is also a differentially expressed protein in KRAS G13D mutations and BRAF V600E mutations, although the specific roles of these differentially expressed proteins, including AHNAK2, were not further explored (94).

### 3.12 Renal cancer

AHNAK is lacking in renal cancer studies while AHNAK2 has been more intensively explored and confirmed to be a pro-oncogenic factor. Wang et al. reported that AHNAK2 expression was elevated in renal clear cell carcinoma and correlated with poorer overall survival (95). Downregulation of AHNAK2 inhibits lipid synthesis and thus affects tumor cell metabolism. According to chromatin immunoprecipitation and dual luciferase reporter gene analysis, HIF1α and the hre1 promoter of AHANK2 bind, and hypoxia-induced upregulation of AHNAK2 expression is dependent on HIF1α. Knockdown of AHNAK2 impairs hypoxia-induced epithelial mesenchymal transition and stem cell properties of tumor cells.

### 3.13 Bladder cancer

In one study, 10 urine samples were collected from patients with bladder urothelial carcinoma (BLCA) and benign urothelial lesion (BUL), and 3 tissue specimens were collected for proteomic analysis. Four candidate BLCA diagnostic proteins including AHNAK, EPPK1, MYH14 and OLFM4 were screened using public databases such as TCGA for joint comparisons. It was further confirmed by cellular immunohistochemistry that only AHNAK2 could discriminate well between BLCA and BUL samples. The expression of AHNAK was also found to be significantly decreased in BLCA compared to BUL, which is consistent with the results in the TCGA database, suggesting that AHNAK may be a suppressor gene and diagnostic marker for bladder cancer (96). However, Okusa et al. reported that AHNAK was more highly expressed in the cytoplasmic membrane of uroepithelial cancer tissues compared to adjacent normal tissues (97). The two studies reached inconsistent conclusions. Lee et al. speculated that the inconsistency might be due to the two different processing performed on samples by immunocytochemistry and immunohistochemistry (96). In another study, a clinical prediction model was constructed for bladder cancer using AHNAK as a factor, which verified that the model is of some predictive value for diagnosis and prognosis (98, 99).

Immunohistochemical analysis was performed in a study and it was found that the sensitivity and specificity of AHNAK2 for identifying severe cystitis with reactive urothelial atypia (RUA) and carcinoma *in situ* (CIS) were 97% and 69% respectively, 97% and 55% between CIS and low-grade invasive bladder cancer, and 80% and 86% between low-grade and high-grade invasive bladder cancer, suggesting that AHNAK2 may be a valuable pathological diagnostic marker for IHC pathology in bladder lesions (100). Koguchi et al. collected pathological specimens from 120 patients who underwent radical resection for bladder cancer for IHC analysis and divided the cases into two groups of high and low AHNAK2 expression based on staining. They found that patients with high AHNAK2 expression had significantly lower recurrence-free survival (RFS) and cancer-specific survival (CSS). Multifactorial analysis showed that high AHNAK2 expression could be an independent risk factor for worse RFS and CSS (101). A similar conclusion was reached in another study in which Komina et al. collected urine samples from 67 cancer patients with bladder occupancy (bladder cancer group N=37, benign bladder tumors N=30) and found that the mean urinary AHNAK2 protein level was almost 10 times higher in bladder cancer patients than that in controls (49.08 pg/mL vs. 5.28 pg/mL) and that AHNAK2 expression level in invasive bladder cancer was significantly higher than that in non-invasive bladder cancer (117.99 pg/mL vs. 7.14 pg/mL). Similarly, AHNAK2 level was significantly higher in muscle-invasive bladder cancer than that in non-muscle-invasive bladder cancer (160.05 pg/mL vs. 23.19 pg/mL) (102). A large sample analysis using the TCGA and GEO databases has been reported to confirm that AHNAK2 is overexpressed in bladder cancer and is associated with poorer overall survival (103).

### 3.14 Prostate cancer

In castration-resistant prostate cancer (CRPC), AHNAK is a downstream target molecule of BRD4, and knockdown of AHNAK or BRD4 inhibits the migration of prostate cancer cells. A decrease in AHNAK expression and cell migration can be seen in treatment of CRPC cells with MZ1, which degrades BRD4. Analysis using the TCGA database revealed a significant correlation between BRD4 and AHNAK mRNA expression with high expression of both being associated with poorer recurrence-free survival (104). Another study reported that AHNAK mutation rate was approximately 4.82% in prostate cancer, ranking it fourth, although it was not studied in depth (105). Studies on the relationship between AHNAK2 and prostate cancer are still lacking.

### 3.15 Tumors in female reproductive system

It was found that AHNAK expression was downregulated in ovarian cancer. Knockdown of AHNAK in ovarian cancer cells was seen to activate the Wnt signaling pathway, and overexpression of AHNAK inhibited cell proliferation, migration, and EMT. The authors hypothesized that AHNAK might regulate ovarian cancer progression through the Wnt pathway (106). The interesting thing is that another report indicated AHNAK overexpression in ovarian cancer was associated with poor prognosis, yet *in vitro*, knockdown of AHNAK inhibited the proliferation, migration, and invasion of CAOV3 and SKOV3 cells in ovarian cancer (107). The authors speculated that this “paradoxical” phenomenon may be related to epigenetic modifications and multiple protein interactions, indicating a complex role of AHNAK proteins in tumor regulation.

A study found by immunohistochemistry and bioinformatic analysis that AHNAK2 was highly expressed in cervical adenocarcinoma and was associated with a poorer prognosis. Moreover, knockdown of AHNAK2 inhibited the value-added and metastasis of cervical cancer Hela cells (108). Using mouse embryonic fibroblasts, Lee et al. confirmed that AHNAK interacts with the receptor-activated Smad (R-Smad, including Smad1, Smad2, and Smad3) and found that it can bind to endogenous Smad3 and regulate the nuclear translocation of Smad3, playing a key role in transforming growth factor-β (TGF-β) induced R-Smad activation (72). Furetermore, AHNAK can induce c-Myc and cell cycle protein D1 downregulation through TGFβ/R-Smad, leading to cervical squamous cell carcinoma cell (siHa) arrest in the G0/G1 phase and inhibition of cell growth (72).

### 3.16 Other malignant tumors

Sudo et al. found that AHNAK was overexpressed in mesothelioma tissues. In the study they examined AHNAK expression in eight mesothelioma cell lines and found that seven mesothelioma cell lines (211H, H28, H226, H2052, H2452, MESO1, and MESO4) had high AHNAK expression, and only MeT-5A had no detectable AHNAK expression. Migration and invasion of the other seven mesothelioma cells were significantly elevated compared to the AHNAK-deficient MeT-5A cell line, although knockdown of AHNAK significantly inhibited the migration and invasion of the seven cells (109).

AHNAK2 mutations in cutaneous squamous cell carcinoma was identified in a study, in which its role was not further explored (110). Saito et al. collected 10 surgically resected specimens of thymic carcinoma and its paraneoplastic tissues for genomic and epigenomic aberration studies. The sequencing revealed an amplification of AHNAK2 located in the region of chromosome 14q32.33, which may be the oncogene of thymic carcinoma, although more in-depth studies are needed to elucidate the specific mechanism (111).

### 3.17 Briefly summarize the signal pathways related to AHNAK family in malignant tumors

**Figure 3:**
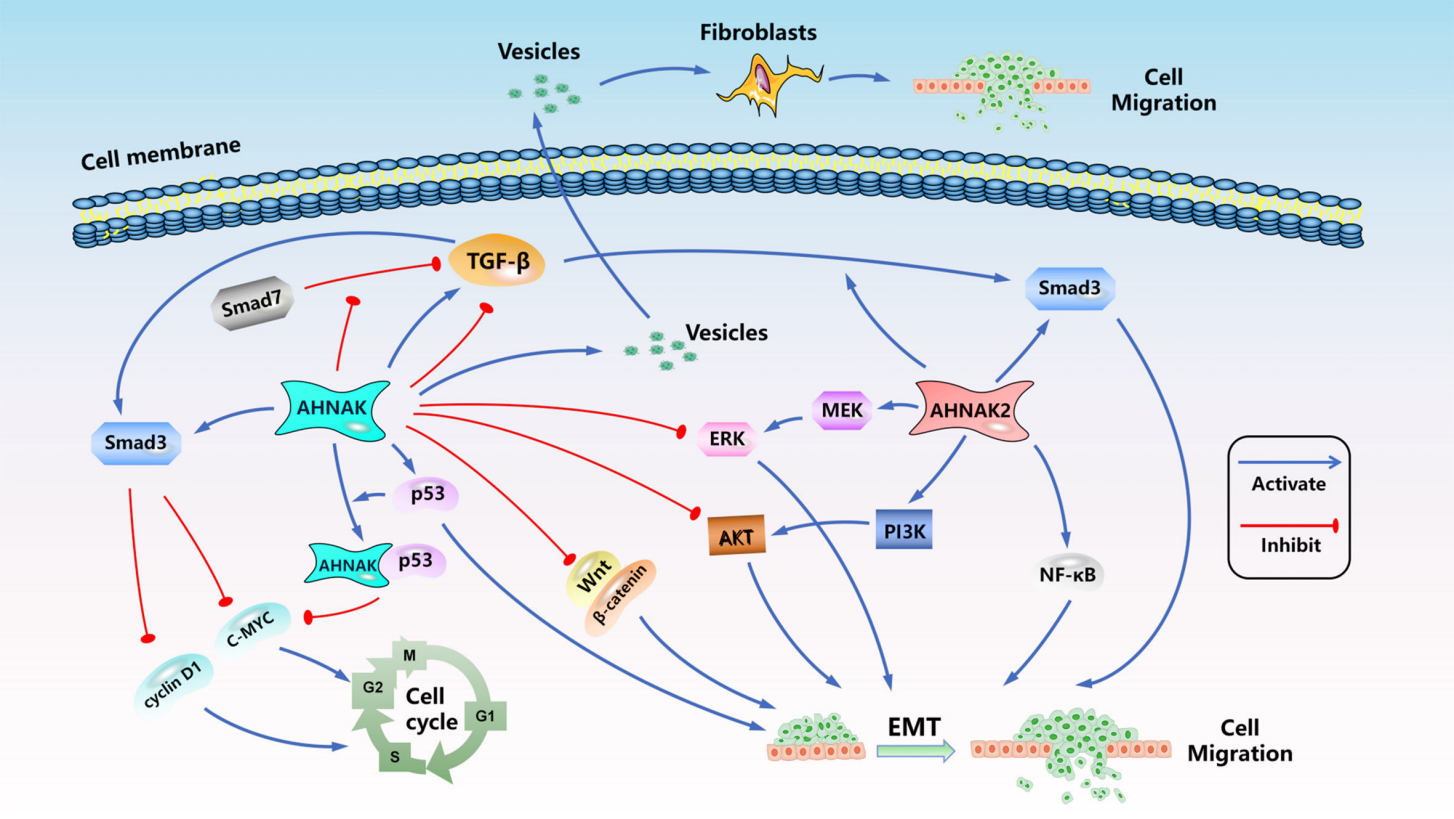
The signal pathways related to AHNAK family in malignant tumors. (
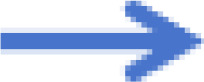
: Activation effect, 
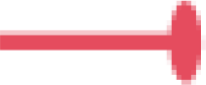
: Inhibition effect).

Hitherto, the exhaustive molecular mechanisms and signaling pathways regulation of AHNAK and AHNAK2 are still in the exploratory stage. One research had pointed out that AHNAK can relieve the inhibitory effect of samd7 on TGF-β, and could inhibit the expression of c-MYC and cyclin D1 by activating the TGF-β/smad3 pathway, resulting in cell cycle arrest (72). AHNAK can also inhibit EMT and migration of tumor cells by inducing inactivation of TGF-β signaling (80). Nevertheless, another study reported that AHNAK is an indispensable factor in tumor cell migration induced by TGF-β (57). TGF-β has two distinct roles in malignancy: tumor suppression based on the induction of growth arrest and apoptosis, and tumor suppression based on the induction of angiogenic capacity and epithelial mesenchymal transition (EMT) (112). AHNAK is closely related to TGF-β. It is speculated that the different functions it exhibits in cancer cells may be related to TGF-β. The results suggested that the regulation of TGF-β signaling pathway by AHNAK seems to be bidirectional. It has been reported that AHNAK2 can promote tumor cell invasion, migration and EMT by activating TGF-β/smad3 signaling (51). At present, there are no reports about bidirectional regulation of TGF-β signal by AHNAK2. P53 plays crucial roles in the regulation of tumor progression, studies have shown that AHNAK could form a complex with P53 and inhibit cell cycle (47). Knockdown AHNAK induced down-regulation of p53 and inhibited tumor cell migration and EMT (82). Silva et al. reported that AHNAK could be transported to extracellular through vesicles and act on fibroblasts, induce fibroblast migration and ECM destruction, and promote tumor progression (74). AHNAK seems to have more inhibitory effect on the regulation of signal pathway. AHNAK could inhibit the signals of Wnt/β-catenin, ERK and AKT, resulting in the decrease of EMT, migration and invasion of tumor cells (75, 88, 93, 106). However, AHNAK2 seems to exist more as an activator of signal pathways, which could promote the activation of PI3K/AKT, MEK/ERK and NF-κB signals and induce EMT, invasion and migration of tumor cells (52, 62, 71). We briefly summarized the AHNAKs-related signal pathways in **Figure 3**. As the AHNAK family, which has been paid more attention recently, its regulatory mechanism in tumor process urgently needs more in-depth research.

## 4 AHNAKs in cancers in bioinformatics database

### 4.1 The expression of AHNAKs in cancers

**Figure 4:**
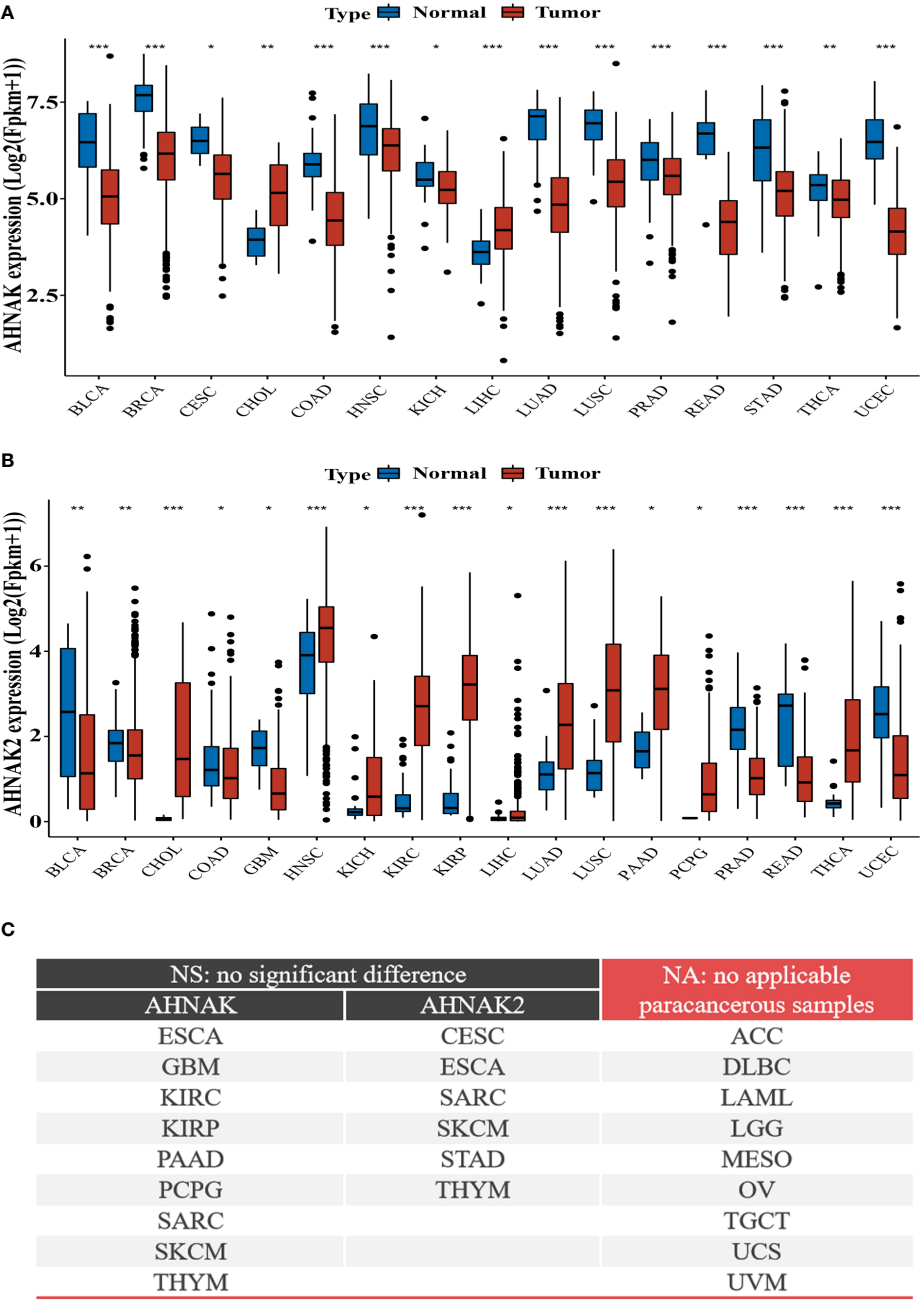
Differential expression of AHNAKs mRNA in tumor and corresponding non-tumor tissues. **(A)** AHNAK downregulation in BLCA, BRCA, CESC, COAD, HNSC, KICH, LUAD, LUSC, PRAD, READ, STAD, THCA, UCEC, and upregulation in CHOL, LIHC. **(B)** AHNAK2 downregulation in BLCA, BRCA, COAD, GBM, PRAD, READ, UCEC and upregulation in CHOL, HNSC, KICH, KIRC, KIRP, LIHC, LUAD, LUSC, PAAD, PCPG, THCA. **(C)** Cancer types with no significant differences or no applicable paracancerous samples. ACC, adrenocortical carcinoma; BLCA, bladder urothelial carcinoma; BRCA, breast invasive carcinoma; CESC, cervical squamous cell carcinoma and endocervical adenocarcinoma; CHOL, cholangiocarcinoma; COAD, colon adenocarcinoma; DLBC, lymphoid neoplasm diffuse large B-cell lymphoma; ESCA, esophageal carcinoma; GBM, glioblastoma multiforme; HNSC, head and neck squamous cell carcinoma; KICH, kidney chromophobe; KIRC, kidney renal clear cell carcinoma; KIRP, kidney renal papillary cell carcinoma; LAML, acute myeloid leukemia; LGG, brain lower grade glioma; LIHC, liver hepatocellular carcinoma; LUAD, lung adenocarcinoma; LUSC, lung squamous cell carcinoma; MESO, mesothelioma; OV, ovarian serous cystadenocarcinoma; PAAD, pancreatic adenocarcinoma; PCPG, pheochromocytoma and paraganglioma; PRAD, prostate adenocarcinoma; READ, rectum adenocarcinoma; SARC, sarcoma; SKCM, skin cutaneous melanoma; STAD, stomach adenocarcinoma; TGCT, testicular germ cell tumors; THCA, thyroid carcinoma; THYM, thymoma; UCEC, uterine corpus endometrial carcinoma; UCS, uterine carcinosarcoma; UVM, uveal melanoma. (NA, no applicable paracancerous samples; NS, no significance; *, p<0.05; **, p<0.01; ***, p<0.001).

**Figure 5:**
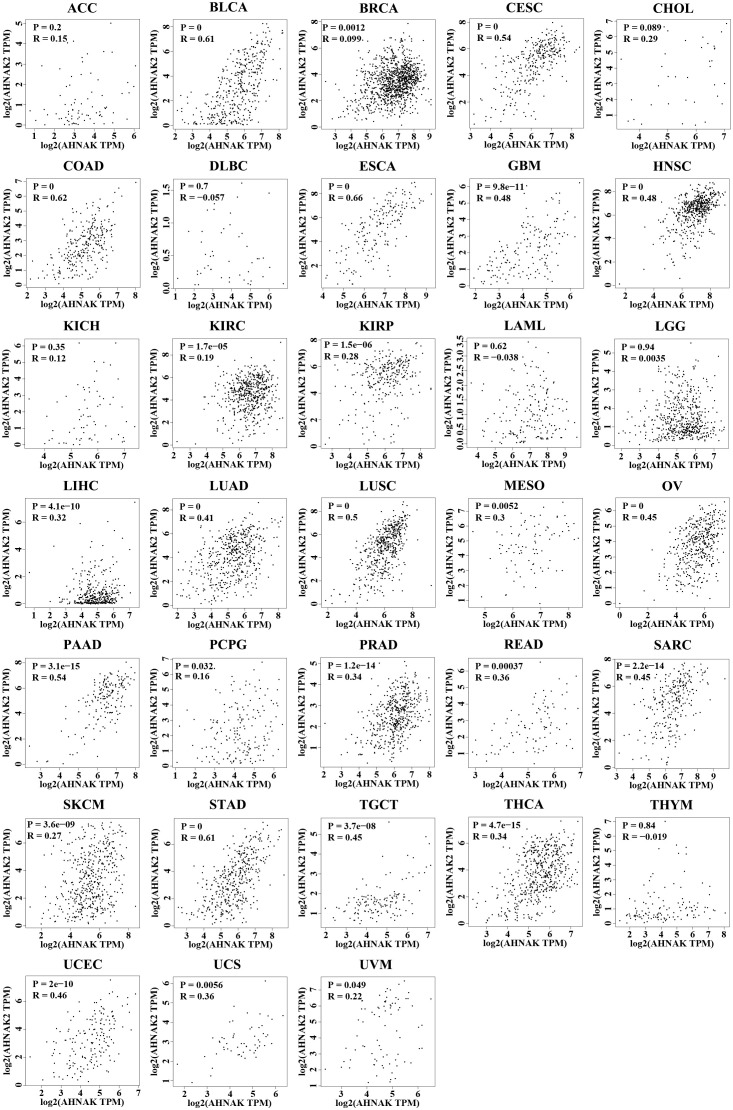
The correlation in mRNA expression between AHNAK and AHNAK2 from different tumor types in the TCGA. (R: Pearson correlation coefficient, p < 0.05 was considered statistically significant).

To further analyze the relationship between AHNAKs and cancers, the mRNA expression and clinical data of 33 cancers in TCGA were collected from UCSC Xena database (https://xenabrowser.net/datapages/). We used R language (4.1.0) to analysis the data. It turned out that AHNAK and AHNAK2 were significantly differentially expressed in cancer and paraneoplastic tissues of some tumors. Among them, AHNAK expression was downregulated in BLCA, BRCA, CESC, COAD, HNSC, KICH, LUAD, LUSC, PRAD, READ, STAD, THCA, UCEC, while upregulated in CHOL and LIHC (**Figure 4A**). For AHNAK2, its expression was downregulated in BLCA, BRCA, COAD, GBM, PRAD, READ and UCEC while up-regulated in CHOL, HNSC, KICH, KIRC, KIRP, LIHC, LUAD, LUSC, PAAD, PCPG and THCA (**Figure 4B**). **Figure 4C** illustrated cancer types with no significant differences or no applicable paracancerous samples. Detailed data on differences in expression between tumor and normal tissues were shown in Table (**Table 1**). Moreover, because of AHNAK and AHNAK2 exhibit roughly 90% homologous sequences, with certain resemblances in their molecular structures and biological functions, we examined the correlation in mRNA expression between AHNAK and AHNAK2 from different tumor types in the TCGA datasets in GEPIA (Gene Expression Profiling Interactive Analysis, http://gepia.cancer-pku.cn/) database. The mRNA expression of AHNAK and AHNAK2 showed significant postive correlation in 27 types of tumors, only 6 types of tumors (ACC, DLBC KICH, LAML, LGG, THYM) showed no significant correlation (**Figure 5**). Interestingly, there was no significant negative correlation in any type of tumor. Detailed correlation data were shown in Table (**Table 2**).

**Table 1:** Differential expression of AHNAKs mRNA in tumor and corresponding non-tumor tissues.

Cancer Type	Number of Cases		AHNAK (Exp: Mean ± SD)				AHNAK2 (Exp: Mean ± SD)			
	Tumor	Normal	Tumor Exp	Normal Exp	logFC	P-Value	Tumor	Normal	logFC	P-Value
ACC	79	–	3.131 ± 0.990	–	–	–	0.457 ± 0.605	–	–	–
BLCA	408	19	4.983 ± 1.047	6.399 ± 0.965	-1.416	<0.001*	1.530 ± 1.420	2.565 ± 1.562	-1.036	0.003*
BRCA	1098	113	6.012 ± 0.948	7.603 ± 0.599	-1.591	<0.001*	1.662 ± 0.902	1.800 ± 0.530	-0.137	0.002*
CESC	306	3	5.500 ± 0.879	6.517 ± 0.678	-1.017	0.046*	2.744 ± 1.288	2.961 ± 1.037	-0.217	0.830
CHOL	36	9	5.058 ± 0.975	3.911 ± 0.486	1.147	0.002*	1.853 ± 1.448	0.069 ± 0.048	1.784	<0.001*
COAD	458	41	4.419 ± 0.953	5.905 ± 0.731	-1.486	<0.001*	1.208 ± 0.870	1.558 ± 1.078	-0.349	0.038*
DLBC	48	–	3.468 ± 1.196	–	–	–	0.139 ± 0.141	–	–	–
ESCA	162	11	5.745 ± 0.838	6.151 ± 1.049	-0.406	0.185	3.326 ± 1.599	2.574 ± 2.209	0.752	0.183
GBM	167	5	3.239 ± 0.921	3.567 ± 0.508	-0.329	0.441	0.878 ± 0.759	1.663 ± 0.654	-0.785	0.017*
HNSC	502	44	6.233 ± 0.878	6.751 ± 0.893	-0.518	<0.001*	4.265 ± 1.226	3.583 ± 1.185	0.683	<0.001*
KICH	65	24	5.229 ± 0.718	5.543 ± 0.698	-0.314	0.045*	0.946 ± 0.971	0.389 ± 0.497	0.557	0.027*
KIRC	531	72	6.066 ± 0.770	6.133 ± 0.567	-0.067	0.517	2.643 ± 1.151	0.474 ± 0.390	2.168	<0.001*
KIRP	289	32	5.588 ± 0.916	5.682 ± 0.470	-0.094	0.861	3.020 ± 1.247	0.545 ± 0.508	2.475	<0.001*
LAML	151	–	6.385 ± 0.991	–	–	–	0.627 ± 0.522	–	–	–
LGG	525	–	4.479 ± 0.937	–	–	–	0.550 ± 0.537	–	–	–
LIHC	373	50	4.171 ± 0.811	3.622 ± 0.509	0.549	<0.001*	0.275 ± 0.558	0.075 ± 0.076	0.200	
LUAD	515	59	4.764 ± 1.115	6.835 ± 0.706	-2.071	<0.001*	2.291 ± 1.270	1.102 ± 0.509	1.189	<0.001*
LUSC	501	49	5.327 ± 0.953	6.833 ± 0.621	-1.506	<0.001*	2.994 ± 1.441	1.202 ± 0.519	1.792	<0.001*
MESO	86	–	6.029 ± 0.703	–	–	–	2.544 ± 1.177	–	–	–
OV	379	–	4.815 ± 0.877	–	–	–	2.106 ± 1.014	–	–	–
PAAD	178	4	5.716 ± 0.796	5.414 ± 0.306	0.302	0.132	2.973 ± 1.245	1.715 ± 0.688	1.258	0.0300*
PCPG	183	3	3.610 ± 0.964	3.665 ± 0.479	-0.054	0.910	0.982 ± 0.946	0.080 ± 0.015	0.902	0.023*
PRAD	496	52	5.516 ± 0.765	5.908 ± 0.748	-0.392	<0.001*	1.095 ± 0.613	2.169 ± 0.857	-1.073	<0.001*
READ	167	10	4.251 ± 0.952	6.505 ± 0.935	-2.254	<0.001*	1.108 ± 0.802	2.401 ± 1.168	-1.293	<0.001*
SARC	263	2	5.642 ± 0.882	5.322 ± 0.866	0.319	0.598	2.557 ± 1.373	1.956 ± 2.402	0.600	0.611
SKCM	471	1	4.794 ± 1.071	5.133	–	–	1.836 ± 1.388	1.020	–	–
STAD	375	32	5.127 ± 0.930	6.2 ± 1.052	-1.073	<0.001*	2.100 ± 1.321	2.828 ± 1.910	-0.728	0.059
TGCT	156	–	4.212 ± 0.970	–	–	–	0.651 ± 0.490	–	–	–
THCA	510	58	4.929 ± 0.756	5.236 ± 0.617	-0.307	0.001*	1.901 ± 1.209	0.439 ± 0.194	1.462	<0.001*
THYM	119	2	3.901 ± 1.326	5.612 ± 2.351	-1.711	0.173	0.447 ± 0.675	0.778 ± 0.812	-0.330	0.309
UCEC	544	35	4.157 ± 0.868	6.439 ± 0.797	-2.282	<0.001*	1.386 ± 1.062	2.507 ± 1.011	-1.121	<0.001*
UCS	56	–	4.041 ± 0.850	–	–	–	1.251 ± 0.630	–	–	–
UVM	80	–	4.321 ± 0.709	–	–	–	2.673 ± 1.444	–	–	–

Exp, expression; SD, Standard Deviation; LogFC, Log2 fold change; -, no applicable datas; *, P-value < 0.05 was considered statistically significant.

**Table 2:** The correlation in mRNA expression between AHNAK and AHNAK2 from different tumor types in the TCGA.

Positive									Negative		
Type	R	P	Type	R	P	Type	R	P	None		
BLCA	0.610	<0.001**	LIHC	0.320	<0.001*	SARC	0.450	<0.001**	Non-correlation		
BRCA	0.099	0.001*	LUAD	0.410	<0.001*	SKCM	0.270	<0.001*	Type	R	P
CESC	0.540	<0.001*	LUSC	0.500	<0.001*	STAD	0.610	<0.001*	ACC	0.15	0.2000
COAD	0.620	<0.001*	MESO	0.300	0.005*	TGCT	0.450	<0.001*	CHOL	0.29	0.089
ESCA	0.620	<0.001*	OV	0.450	<0.001*	THCA	0.340	<0.001*	DLBC	-0.057	0.700
GBM	0.480	<0.001*	PAAD	0.540	<0.001**	UCEC	0.460	<0.001*	KICH	0.12	0.350
HNSC	0.480	<0.001*	PCPG	0.160	0.032*	UCS	0.360	0.006*	LAML	-0.038	0.620
KIRC	0.190	<0.001*	PRAD	0.340	<0.001**	UVM	0.220	0.049*	LGG	0.004	0.940
KIRP	0.280	<0.001*	READ	0.360	<0.001*				THYM	-0.019	0.840

R: Pearson correlation coefficient, *: p < 0.05 was considered statistically significant.

**Figure 6:**
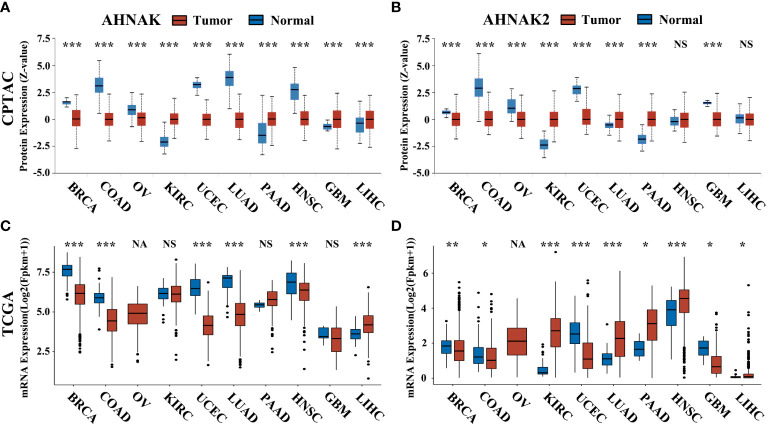
The protein expression of AHNAK and AHNAK2 in CPTAC and the corresponding mRNA expression in TCGA. **(A, B)** The AHNAK or AHNAK2 protein expression of eight cancers in CPTAC. **(C, D)** The AHNAK or AHNAK2 mRNA expression of the eight cancers in TCGA. (NA, no applicable paracancerous samples; NS, no significance; *, p<0.05; **, p<0.01; ***, p<0.001).

In addition, we analyezed the protein expression of AHNAK and AHNAK2 in CPTAC through UALCAN database (https://ualcan.path.uab.edu/). In the CPTAC database of UALCAN, 8 types of tumors have relatively complete comparative data of protein expression in tumor and normal samples. Among them, AHNAK expression was downregulated in BRCA, COAD, OV, UCEC, LUAD and HNSC, while upregulated in KIRC, PAAD, GBM and LIHC (**Figure 6A**). For AHNAK2, its expression was downregulated in BRCA, COAD, OV and GBM, while upregulated in KIRC, PAAD, LUAD and PAAD (**Figure 6B**). Understanding the correlation between protein abundance and RNA level is crucial, so we performed simultaneous comparisons. The protein and mRNA expression of AHNAK had different trends in KIRC and GBM, while AHNAK2 was not consistent only in LIHC (**Figure 6**). The results revealed that the protein and mRNA levels of AHNAK and AHNAK2 were highly consistent.

**Figure 7:**
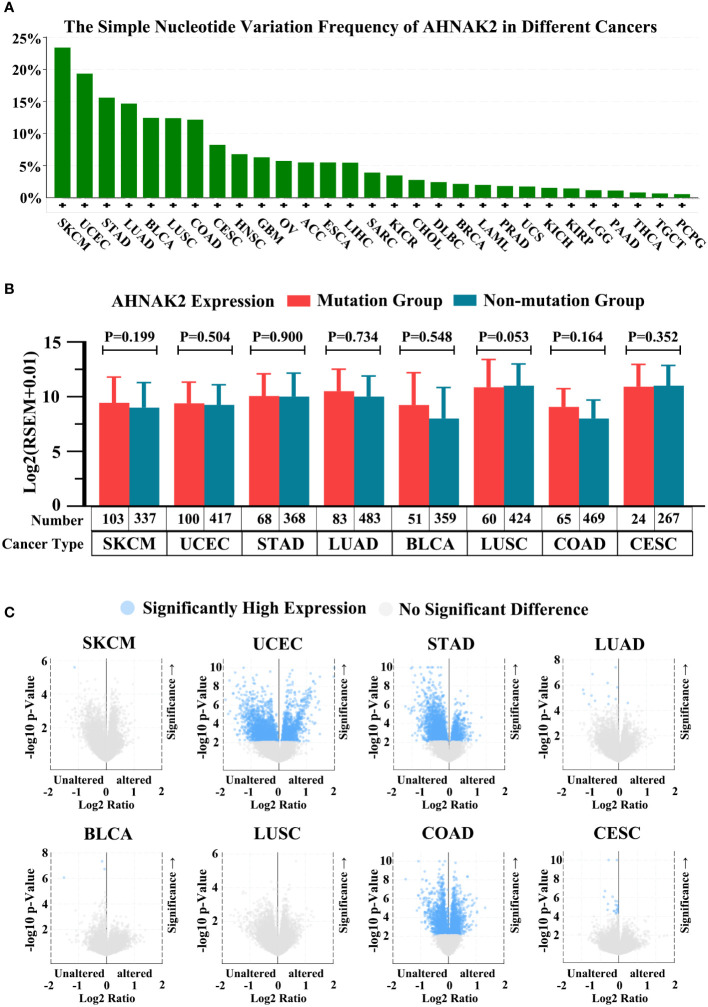
Analysis of AHNAK2 mutations in cancers. **(A)** The simple nucleotide variation frequency of AHNAK2 in different cancers in TCGA. **(B)** The mRNA expression of AHNAK2 in mutation groups and non-mutation groups. **(C)** Comparison of mRNA expression of other genes in AHNAK2 mutation groups and non-mutation groups. (p < 0.05 was considered statistically significant).

In present studies reported AHNAK2 is among the most frequently mutated genes in several tumor types. It is common knowledge that gene mutations may affect its expression levels, consequently, we analyzed the simple nucleotide variation frequency in different cancers and the mRNA expression of AHNAK2 between mutation and not-mutation samples in TCGA in cBioPortal database (https://www.cbioportal.org/). As shown in **Figure 7**, AHNAK2 gene mutations were found in a variety of tumors, and the mutation frequency in SKCM was as high as 24% (**Figure 7A**). We selected the top eight cancers for subsequent analyses, including SKCM, UCEC, STAD, LUAD, BLCA, LUSC, COAD and CESC in turn. Confusingly, AHNAK2 mutation did not seem to have a significant effect on mRNA expression (**Figure 7B**). Of course, due to the limitations of the sample and other reasons, more research is needed in the future. Moreover, We also analyzed the expression of other genes after the mutation of AHNAK2, the brief results are shown in **Figure 7** in the form of a volcano map and the detailed results can be seen in the **Supplementary Material** (**Figure 7C**, **Supplementary Excel**). Due to the lack of in-depth researches, the upstream and downstream genes of AHNAK2 were not yet fully determined, so we have not further analyzed and discussed the results.

### 4.2 AHNAKs and prognosis

Using univariate regression analysis, we examined the associations of AHNAK and AHNAK2 with the prognosis of 33 cancers by R language (**Tables 3**, **4**). It is worth proposing that AHNAK2 was significantly associated with all four indicators including OS, PFS, DFS and DSS in lung adenocarcinoma patients, which is also consistent with our previous findings (50).

**Table 3:** Contribution of the expression of AHNAK to survival by univariate Cox regression analysis in cancers.

Cancer Type	OS			PFS			DFS			DSS		
	HR	95%CI	P	HR	95%CI	P	HR	95%CI	P-value	HR	95%CI	P
ACC	1.129	0.750-1.698	0.561	1.170	0.847-1.617	0.341	1.210	0.636-2.300	0.561	1.091	0.715-1.665	0.686
BLCA	1.380	1.180-1.615	0.000*	1.290	1.103-1.508	0.001*	0.956	0.667-1.371	0.807	1.442	1.188-1.750	0.000*
BRCA	0.980	0.827-1.160	0.812	0.941	0.793-1.117	0.489	0.857	0.690-1.064	0.162	0.870	0.699-1.082	0.211
CESC	1.335	0.997-1.787	0.053	1.068	0.810-1.408	0.642	0.886	0.567-1.383	0.593	1.278	0.918-1.780	0.146
CHOL	0.874	0.541-1.411	0.582	0.953	0.601-1.511	0.837	1.411	0.672-2.960	0.363	0.947	0.567-1.583	0.836
COAD	1.049	0.844-1.304	0.667	1.064	0.885-1.279	0.508	1.095	0.736-1.630	0.653	1.058	0.819-1.366	0.667
DLBC	1.236	0.672-2.275	0.495	1.280	0.763-2.148	0.350	1.278	0.512-3.192	0.599	1.232	0.487-3.115	0.659
ESCA	0.720	0.542-0.957	0.024*	0.794	0.614-1.027	0.079	0.940	0.569-1.551	0.807	0.742	0.534-1.032	0.076
GBM	1.121	0.949-1.325	0.179	1.113	0.930-1.331	0.243	–	–	–	1.143	0.954-1.370	0.147
HNSC	1.036	0.879-1.221	0.676	0.911	0.771-1.075	0.270	0.918	0.580-1.453	0.716	1.011	0.817-1.251	0.921
KICH	0.774	0.320-1.871	0.569	0.665	0.303-1.458	0.309	0.697	0.151-3.215	0.643	0.859	0.312-2.367	0.769
KIRC	0.757	0.641-0.894	0.001*	0.837	0.698-1.003	0.054	1.117	0.634-1.969	0.702	0.708	0.579-0.867	0.001*
KIRP	0.930	0.664-1.302	0.672	0.690	0.530-0.898	0.006*	0.743	0.500-1.104	0.141	0.685	0.469-1.001	0.051
LAML	1.142	0.926-1.409	0.214	–	–	–	–	–	–	–	–	–
LGG	0.880	0.733-1.057	0.172	0.929	0.801-1.079	0.335	0.978	0.618-1.546	0.923	0.937	0.771-1.139	0.512
LIHC	0.966	0.777-1.202	0.758	0.989	0.816-1.198	0.910	0.860	0.693-1.068	0.172	0.939	0.707-1.248	0.665
LUAD	1.091	0.958-1.242	0.190	1.042	0.922-1.176	0.511	0.957	0.798-1.147	0.632	1.103	0.936-1.301	0.241
LUSC	1.069	0.919-1.244	0.387	1.188	0.982-1.437	0.077	0.946	0.714-1.254	0.699	1.141	0.894-1.456	0.290
MESO	1.156	0.803-1.665	0.436	1.097	0.755-1.594	0.628	0.791	0.342-1.827	0.583	1.068	0.670-1.701	0.783
OV	1.142	0.985-1.324	0.079	0.957	0.837-1.093	0.514	0.912	0.751-1.106	0.349	1.166	0.994-1.367	0.059
PAAD	1.743	1.287-2.36	0.000*	1.701	1.284-2.252	0.000*	2.870	1.409-5.846	0.004*	1.775	1.261-2.499	0.001*
PCPG	1.006	0.489-2.069	0.988	0.951	0.618-1.464	0.820	1.523	0.554-4.186	0.414	1.178	0.504-2.754	0.706
PRAD	0.669	0.333-1.341	0.257	0.779	0.602-1.008	0.057	0.779	0.488-1.242	0.294	0.438	0.157-1.220	0.114
READ	0.769	0.490-1.209	0.255	1.004	0.685-1.471	0.985	1.290	0.425-3.911	0.653	0.528	0.293-0.951	0.034*
SARC	0.758	0.594-0.967	0.026*	0.777	0.636-0.949	0.014*	0.865	0.651-1.148	0.316	0.654	0.499-0.858	0.002*
SKCM	0.981	0.863-1.114	0.763	0.955	0.858-1.063	0.401	–	–	–	0.937	0.818-1.073	0.346
STAD	1.113	0.929-1.333	0.244	1.105	0.910-1.343	0.313	1.149	0.813-1.623	0.431	1.122	0.893-1.411	0.324
TGCT	1.619	0.528-4.959	0.399	1.074	0.787-1.464	0.654	0.971	0.695-1.358	0.865	1.792	0.524-6.127	0.352
THCA	1.224	0.619-2.421	0.561	0.908	0.639-1.291	0.591	1.076	0.658-1.759	0.770	0.707	0.275-1.817	0.472
THYM	1.019	0.617-1.683	0.941	0.912	0.652-1.276	0.591	–	–	–	0.644	0.262-1.583	0.338
UCEC	0.984	0.777-1.247	0.895	0.917	0.749-1.124	0.405	0.846	0.628-1.141	0.274	1.039	0.779-1.386	0.793
UCS	0.823	0.574-1.180	0.289	0.773	0.542-1.102	0.155	0.572	0.281-1.166	0.124	0.708	0.481-1.043	0.081
UVM	0.686	0.344-1.365	0.283	0.922	0.509-1.670	0.789	–	–	–	0.652	0.314-1.353	0.251

OS, Overall survival, PFS, Progression free survival, DFS, Disease free survival, DSS, Disease specific survival. *, P-value < 0.05 was considered statistically significant.

**Table 4:** Contribution of the expression of AHNAK2 to survival by univariate Cox regression analysis in cancers.

Cancer Type	OS			PFS			DFS			DSS		
	HR	95%CI	P	HR	95%CI	P	HR	95%CI	P-value	HR	95%CI	P
ACC	0.932	0.475-1.826	0.837	0.825	0.463-1.470	0.514	1.108	0.400-3.068	0.844	0.725	0.319-1.647	0.443
BLCA	1.210	1.096-1.336	0.000*8**	1.193	1.079-1.319	0.001*	1.151	0.908-1.459	0.244	1.281	1.138-1.442	0.000*
BRCA	1.070	0.889-1.289	0.473	1.282	1.078-1.525	0.005*	1.361	1.096-1.690	0.005*	1.206	0.950-1.530	0.124
CESC	1.110	0.924-1.334	0.264	0.977	0.815-1.171	0.799	0.806	0.598-1.087	0.158	1.123	0.908-1.390	0.285
CHOL	1.115	0.811-1.532	0.504	1.102	0.816-1.489	0.527	1.239	0.759-2.021	0.391	1.142	0.816-1.598	0.440
COAD	1.165	0.940-1.445	0.163	1.344	1.119-1.614	0.002*	1.435	0.949-2.169	0.087	1.356	1.047-1.756	0.021*
DLBC	2.853	0.019-418.4	0.680	0.001	0.000-3.901	0.097	0.014	0-30614.376	0.565	0.005	0.0-2540.95	0.426
ESCA	0.887	0.761-1.035	0.128	0.963	0.842-1.102	0.585	1.111	0.853-1.447	0.433	0.865	0.721-1.038	0.119
GBM	1.147	0.948-1.388	0.160	1.277	1.060-1.539	0.010*	–	–	–	1.173	0.962-1.430	0.114
HNSC	1.071	0.952-1.204	0.256	0.989	0.880-1.112	0.857	0.871	0.639-1.187	0.381	1.054	0.908-1.225	0.488
KICH	1.166	0.574-2.370	0.671	1.200	0.625-2.304	0.584	1.423	0.527-3.844	0.487	1.086	0.473-2.491	0.846
KIRC	1.096	0.961-1.250	0.172	0.981	0.857-1.124	0.786	0.977	0.633-1.507	0.915	1.074	0.910-1.267	0.397
KIRP	1.032	0.802-1.328	0.806	0.873	0.709-1.074	0.199	0.842	0.630-1.127	0.248	0.931	0.691-1.253	0.636
LAML	1.342	0.925-1.947	0.121	–	–	–	–	–	–	–	–	–
LGG	1.251	0.968-1.617	0.087	1.217	0.965-1.535	0.097	0.429	0.136-1.356	0.149	1.237	0.940-1.626	0.128
LIHC	1.061	0.790-1.425	0.695	1.016	0.785-1.315	0.902	0.948	0.690-1.303	0.743	1.114	0.783-1.585	0.550
LUAD	1.305	1.156-1.472	0.000*	1.222	1.094-1.365	0.000*	1.260	1.068-1.485	0.006*	1.347	1.156-1.571	0.000*
LUSC	0.997	0.903-1.101	0.955	1.051	0.933-1.185	0.410	0.911	0.763-1.089	0.308	1.040	0.892-1.212	0.618
MESO	1.244	1.027-1.506	0.025*	1.179	0.961-1.446	0.115	1.556	0.806-3.003	0.188	1.308	1.022-1.674	0.033*
OV	1.180	1.042-1.335	0.009*	0.996	0.886-1.119	0.944	0.984	0.827-1.171	0.855	1.185	1.035-1.356	0.014*
PAAD	1.479	1.236-1.772	0.000*	1.401	1.187-1.654	0.000*	1.904	1.295-2.799	0.001*	1.473	1.205-1.800	0.000*
PCPG	0.381	0.102-1.427	0.152	0.795	0.490-1.289	0.352	0.958	0.356-2.583	0.933	0.218	0.027-1.764	0.153
PRAD	0.884	0.326-2.398	0.808	0.658	0.463-0.935	0.019*	0.450	0.235-0.861	0.016*	0.399	0.063-2.537	0.330
READ	1.296	0.832-2.018	0.251	1.318	0.898-1.934	0.158	1.268	0.465-3.456	0.643	0.963	0.487-1.903	0.913
SARC	0.835	0.720-0.969	0.017*	0.868	0.766-0.983	0.026*	0.857	0.716-1.026	0.094	0.818	0.695-0.963	0.016*
SKCM	1.115	1.017-1.221	0.020*	1.035	0.956-1.120	0.393	–	–	–	1.070	0.967-1.184	0.188
STAD	1.097	0.975-1.235	0.125	1.152	1.016-1.306	0.028*	1.158	0.931-1.440	0.186	1.139	0.980-1.323	0.090
TGCT	0.067	0.000-15.07	0.328	1.168	0.625-2.183	0.626	0.964	0.486-1.912	0.917	0.008	0.000-13.35	0.203
THCA	1.139	0.762-1.703	0.526	1.316	1.059-1.636	0.013*	1.258	0.930-1.702	0.137	1.000	0.538-1.858	0.999
THYM	1.308	0.577-2.965	0.520	1.427	0.889-2.291	0.141	–	–	–	0.501	0.033-7.715	0.620
UCEC	1.264	1.056-1.513	0.011*	1.054	0.892-1.245	0.539	0.933	0.717-1.214	0.606	1.378	1.116-1.700	0.003*
UCS	0.821	0.464-1.452	0.497	0.775	0.456-1.318	0.347	0.774	0.283-2.115	0.617	0.819	0.462-1.452	0.495
UVM	1.937	1.344-2.791	0.000*	1.969	1.410-2.751	0.000**	–	–	–	2.091	1.404-3.113	0.000*

OS, Overall survival; PFS, Progression free survival; DFS, Disease free survival; DSS, Disease specific survival. *, P-value < 0.05 was considered statistically significant.

### 4.3 Functional Enrichment of AHNAKs in TCGA database

We also respectively screened the 50 genes most closely associated with AHNAK or AHNAK2 expression in GEPIA using data from tumor tissue specimens of 33 cancers in TCGA, in order to explore the function of AHNAKs (**Table 5**). Gene Ontology analysis was performed by virtue of DAVID Bioinformatics Resources (containing three parts: Biological Process, BP; Cellular Component, CC; Molecular Function, MF, with the top 10 enriched functions for each section, https://david.ncifcrf.gov/). We found that AHNAK has a relatively complex functional enrichment, including Wnt, MAPK/AKT signaling pathway, cell adhesion, membrane repair, calcium channels and other related (**Table 6**). In contrast, AHNAK2 function was relatively concentrated, mainly related to cell adhesion, cytoskeleton structure and calcium channel regulation (**Table 7**).

**Table 5:** AHNAKs expression related genes.

AHNAK expression related genes (Top 50)			AHNAK2 expression related genes (Top 50)		
Gene Symbol	Gene ID	PCC	Gene Symbol	Gene ID	PCC
ATL3	ENSG00000184743.12	0.6	KRT6A	ENSG00000205420.10	0.58
LRRFIP1	ENSG00000124831.18	0.6	GJB3	ENSG00000188910.7	0.58
TMOD3	ENSG00000138594.12	0.6	PKP1	ENSG00000081277.11	0.58
HIPK3	ENSG00000110422.11	0.58	GJB5	ENSG00000189280.3	0.58
SLAIN2	ENSG00000109171.14	0.58	KRT5	ENSG00000186081.11	0.58
SP1	ENSG00000185591.9	0.58	BICD2	ENSG00000185963.13	0.57
ELMSAN1	ENSG00000156030.12	0.56	PERP	ENSG00000112378.11	0.57
MAP3K2	ENSG00000169967.16	0.56	TUBA4A	ENSG00000127824.13	0.56
MYOF	ENSG00000138119.16	0.56	MICALL1	ENSG00000100139.13	0.56
SLK	ENSG00000065613.13	0.56	CLCA2	ENSG00000137975.7	0.56
TRIP11	ENSG00000100815.12	0.56	LAMB3	ENSG00000196878.12	0.56
AP4E1	ENSG00000081014.10	0.55	DSC3	ENSG00000134762.16	0.56
C16orf72	ENSG00000182831.11	0.55	FAM83G	ENSG00000188522.14	0.56
FBXW2	ENSG00000119402.16	0.55	SERPINB5	ENSG00000206075.13	0.55
CSNK1G1	ENSG00000169118.15	0.54	DSG3	ENSG00000134757.4	0.55
MFAP3	ENSG00000037749.11	0.54	SFN	ENSG00000175793.11	0.55
SEC24B	ENSG00000138802.11	0.54	DSP	ENSG00000096696.13	0.54
CSNK1A1	ENSG00000113712.16	0.53	TRIM29	ENSG00000137699.16	0.54
SPOPL	ENSG00000144228.8	0.53	KCTD11	ENSG00000213859.4	0.54
SUSD6	ENSG00000100647.7	0.53	KRT14	ENSG00000186847.5	0.54
WASF2	ENSG00000158195.10	0.53	TLDC1	ENSG00000140950.15	0.53
BTBD7	ENSG00000011114.14	0.52	ANXA2	ENSG00000182718.16	0.53
CNOT6L	ENSG00000138767.12	0.52	FAT2	ENSG00000086570.12	0.53
KLF7	ENSG00000118263.14	0.52	BNC1	ENSG00000169594.12	0.53
LUZP1	ENSG00000169641.13	0.52	COL17A1	ENSG00000065618.16	0.52
MARK2	ENSG00000072518.20	0.52	PKP3	ENSG00000184363.9	0.52
MTF1	ENSG00000188786.9	0.52	TUBB6	ENSG00000176014.12	0.52
SPTY2D1	ENSG00000179119.14	0.52	ANXA2P2	ENSG00000231991.4	0.52
TRIP12	ENSG00000153827.13	0.52	DUSP7	ENSG00000164086.9	0.52
VPS4B	ENSG00000119541.9	0.52	LAMC2	ENSG00000058085.14	0.52
EFCAB14	ENSG00000159658.10	0.51	KRT17	ENSG00000128422.15	0.51
JAK1	ENSG00000162434.11	0.51	CDH3	ENSG00000062038.13	0.51
MYH9	ENSG00000100345.20	0.51	ADGRF4	ENSG00000153294.11	0.51
STRN	ENSG00000115808.11	0.51	KLC3	ENSG00000104892.16	0.5
TMEM127	ENSG00000135956.8	0.51	FAM83B	ENSG00000168143.8	0.5
UBN1	ENSG00000118900.14	0.51	CERS3	ENSG00000154227.13	0.5
ZFP91	ENSG00000186660.14	0.51	IL20RB	ENSG00000174564.12	0.5
AFF4	ENSG00000072364.12	0.5	RP3-523K23.2	ENSG00000261116.1	0.5
ASXL2	ENSG00000143970.16	0.5	TENM2	ENSG00000145934.15	0.5
CAB39	ENSG00000135932.10	0.5	SLC2A1	ENSG00000117394.19	0.49
CACUL1	ENSG00000151893.14	0.5	DSC2	ENSG00000134755.14	0.49
FKBP15	ENSG00000119321.8	0.5	GJB2	ENSG00000165474.5	0.49
GAPVD1	ENSG00000165219.21	0.5	ITGA6	ENSG00000091409.14	0.49
MYO9A	ENSG00000066933.15	0.5	GSDMC	ENSG00000147697.8	0.48
NUMB	ENSG00000133961.19	0.5	LAD1	ENSG00000159166.13	0.48
RIC1	ENSG00000107036.11	0.5	LAMA3	ENSG00000053747.15	0.48
ZDHHC5	ENSG00000156599.10	0.5	FSCN1	ENSG00000075618.17	0.48
ADAM10	ENSG00000137845.14	0.49	FAM83A	ENSG00000147689.16	0.48
CAST	ENSG00000153113.23	0.49	IFFO2	ENSG00000169991.10	0.48
CTNND1	ENSG00000198561.12	0.49	S100A2	ENSG00000196754.10	0.48

PCC, Pearson Correlation Coefficient.

**Table 6:** Gene ontology analysis of AHNAK by DAVID Bioinformatics Resources.

Category	Term	Count	P-Value	FDR
BP	peptidyl-serine phosphorylation	5	0.001	0.150
BP	Wnt signaling pathway	5	0.001	0.150
BP	protein phosphorylation	7	0.002	0.150
BP	plasma membrane repair	3	0.002	0.150
BP	endocytosis	4	0.015	0.940
BP	intracellular signal transduction	5	0.023	1.000
BP	interleukin-15-mediated signaling pathway	2	0.026	1.000
BP	actin filament-based movement	2	0.040	1.000
BP	Golgi organization	3	0.040	1.000
BP	RPSMNRL	2	0.049	1.000
CC	cytosol	23	0.002	0.200
CC	cytoplasm	23	0.003	0.200
CC	adherens junction	4	0.008	0.380
CC	membrane	16	0.011	0.390
CC	nucleus	22	0.013	0.390
CC	early endosome	4	0.032	0.700
CC	cytoskeleton	5	0.034	0.700
CC	growth cone	3	0.038	0.700
CC	glutamatergic synapse	4	0.064	0.88
CC	macromolecular complex	5	0.066	0.88
MF	cadherin binding	9	0.000	0.000
MF	protein serine/threonine/tyrosine kinase activity	7	0.001	0.028
MF	protein serine/threonine kinase activity	6	0.002	0.079
MF	actin binding	5	0.009	0.180
MF	ATP binding	10	0.009	0.180
MF	protein kinase activity	5	0.012	0.200
MF	protein binding	38	0.017	0.230
MF	protein homodimerization activity	6	0.030	0.340
MF	small GTPase binding	4	0.032	0.340
MF	histone acetyltransferase binding	2	0.053	0.520

BP, Biological Process; CC, Cellular Component; MF, Molecular Function. RPSMNRL, regulation of postsynaptic specialization membrane neurotransmitter receptor levels.

**Table 7:** Gene ontology analysis of AHNAK2 by DAVID Bioinformatics Resources.

Category	Term	Count	P-Value	FDR
BP	cell-cell adhesion	14	0.000	0.000
BP	epidermis development	10	0.000	0.000
BP	intermediate filament organization	5	0.000	0.002
BP	cell adhesion	9	0.000	0.003
BP	keratinization	5	0.000	0.003
BP	system development	3	0.000	0.006
BP	cell-cell junction assembly	4	0.000	0.006
BP	HCAVPMAM	5	0.001	0.024
BP	cornification	3	0.001	0.024
BP	tissue development	3	0.003	0.080
CC	cornified envelope	10	0.000	0.000
CC	desmosome	7	0.000	0.000
CC	adherens junction	9	0.000	0.000
CC	cell-cell junction	8	0.000	0.000
CC	intermediate filament	7	0.000	0.000
CC	basement membrane	6	0.000	0.000
CC	cell junction	7	0.000	0.000
CC	extracellular exosome	16	0.000	0.001
CC	connexin complex	3	0.001	0.013
CC	keratin filament	4	0.002	0.019
MF	structural constituent of cytoskeleton	6	0.000	0.000
MF	cadherin binding	8	0.000	0.000
MF	calcium ion binding	10	0.000	0.002
MF	structural molecule activity	6	0.000	0.002
MF	cadherin binding involved in cell-cell adhesion	3	0.001	0.015
MF	gap junction channel activity	3	0.001	0.019
MF	structural constituent of epidermis	3	0.003	0.042
MF	extracellular matrix structural constituent	4	0.005	0.049
MF	phosphatidylserine binding	3	0.010	0.095
MF	CAPBIBHCPMC	2	0.012	0.095

BP, Biological Process; CC, Cellular Component; MF, Molecular Function. HCAVPMAM, homophilic cell adhesion via plasma membrane adhesion molecules; CAPBIBHCPMC, cell adhesive protein binding involved in bundle of His cell-Purkinje myocyte communication.

## 5 Conclusion and prospect

Given all that, the AHNAK family has a variety of important biological functions, such as calcium channel regulation, barrier formation, and cytoskeleton and cell adhesion regulation, and plays an important part in the development of malignant tumors. The AHNAK family mediates the regulation of tumor cell migration and invasion through various pathways. From the current studies, it appears that AHNAK may play different roles in different tumors, even though within the same tumor its role is controversial, such as TNBC, melanoma and bladder cancer. Unfortunately, the role of AHNAK in all cancer types reported in three or more studies has been somewhat controversial. However, AHNAK2 seems more likely to act as an oncogenic gene. The aberrant overexpression of AHNAK2 has been consistently recognized to promote the progression of lung adenocarcinoma, thyroid cancer, and bladder cancer (with at least three reports for each type). However, as a tumor-related huge protein family that has only been highlighted in recent years, in-depth molecular mechanistic studies on this family, especially regarding the regulation of EMT and downstream signaling pathways such as P53, MAPK/AKT, TGF-β by the AHNAK family are lacking and need to be further explored in the future with a view to providing new targets for tumor diagnosis and treatment.

## Author contributions

SZ: Conceptualization, Data curation, Funding acquisition, Writing – original draft. ZC: Conceptualization, Supervision, Writing – review & editing. HL: Conceptualization, Supervision, Writing – review & editing.

## Glossary

**Table:**

ACC	adrenocortical carcinoma
AF	atrial fibrillation
AKT	protein kinase B
AR-CMT	autosomal recessive CMT
AUC	area under curve
BLCA	bladder urothelial carcinoma
BLCA	bladder urothelial carcinoma
BMP2	human hone morphogenetic protein 2
BMP4	human hone morphogenetic protein 4
BP	biological Process
BRCA	breast invasive carcinoma
BRD4	bromodomain containing 4
BUL	benign urothelial lesion
CAD	coronary artery disease
Cavβ2	the β2 subunit of cardiomyocyte L type calcium channels
Cavβ4	the β4 subunit of cardiomyocyte L-type calcium channels
CC	cellular component
CESC	cervical squamous cell carcinoma and endocervical adenocarcinoma
CHOL	cholangiocarcinoma
CIS	carcinoma in situ
CMT	charcot marie tooth
COAD	colon adenocarcinoma
CRPC	castration-resistant prostate cancer
CRU	center recall unit
CSS	cancer-specific survival
DFS	disease free survival
DLBC	lymphoid neoplasm diffuse large B-cell lymphoma
DSS	disease specific survival
EBV	epstein barr virus
EMT	epithelial mesenchymal transition
EPPK1	Human Epiplakin 1
ERK	extracellular regulated protein kinases
ESCA	esophageal carcinoma
FGF1	fibroblast growth factor 1
FGF21	fibroblast growth factor 21
GATA4	GATA binding protein 4
GBM	glioblastoma
GBM	glioblastoma multiforme
GC	gastric cancer
GEO	gene omnibus
GO	gene ontology
HCC	hepatocellular carcinoma
HIF1α	hypoxia inducible factor-1α
HNSC	head and neck squamous cell carcinoma
IDH	isocitrate dehydrogenase
IGF-1R	interact with insulin-like growth factor 1 receptor
IHC	immunohistochemistry
KICH	kidney chromophobe
KIRC	kidney renal clear cell carcinoma
KIRP	kidney renal papillary cell carcinoma
LAML	acute myeloid leukemia
LGG	brain lower grade glioma
LIHC	liver hepatocellular carcinoma
LRP1B	low density lipoprotein receptor-related protein 1B
LUAD	lung adenocarcinoma
LUSC	lung squamous cell carcinoma
LVGCC	type voltage gated calcium channels
MAPK	mitogen-activated protein kinase
MESO	mesothelioma
MF	molecular function
MMTV	mouse mammary tumor virus
MYH14	myosin heavy chain 14
NAL	neoantigen load levels
NF-κB	nuclear factor kappa B
OLFM4	Olfactomedin-4
OS	overall survival
OV	ovarian serous cystadenocarcinoma
PAAD	pancreatic adenocarcinoma
PBMC	peripheral blood mononuclear cells
PCPG	pheochromocytoma and paraganglioma
PDAC	pancreatic ductal adenocarcinoma
PFS	progression free survival
PI3K	Phosphatidylinositol 3 hydroxykinase
PIK3CA	phosphatidylinositol 3-kinase catalytic alpha
PKA	protein kinase A
PRAD	prostate adenocarcinoma
PRX	Periaxin
PTC	papillary thyroid carcinoma
PyVT	Polyoma Virus middle T antigen
READ	rectum adenocarcinoma
RFS	recurrence-free survival
RNF38	ring finger protein 38
RPL	recurrent pregnancy loss
RSI	radiation sensitivity index
RUA	reactive urothelial atypia
SARC	sarcoma
SKCM	skin cutaneous melanoma
ST2	tumorigenicity 2
STAD	stomach adenocarcinoma
TCGA	The Cancer Genome Atlas
TGCT	testicular germ cell tumors
TGF-β	transforming growth factor β
THCA	thyroid carcinoma
THYM	thymoma
TIIC	tumor-infiltrating immune cell
TMB	tumor mutational burden
TNBC	triple-negative breast cancer
UBE3C	ubiquitin protein ligase E3C
UCEC	uterine corpus endometrial carcinoma
UCS	uterine carcinosarcoma
UM	uveal melanoma
UVM	uveal melanoma
