# Supplementary material for: AHNAKs roles in physiology and malignant tumors
Source: Front Oncol. 2023 Nov 14;13:1258951. doi: 10.3389/fonc.2023.1258951 (PMC10682155; doi:10.3389/fonc.2023.1258951)
Supplement: Supplementary file 1 [file Table_1.docx]

Table S1: Comparison of the role of AHNAK and AHNAK2 in different cancer subtypes

| Lung cancer | AHNAK | AHNAK2 |
| --- | --- | --- |
| Lung adenocarcinoma | bi-directional regulation | Carcinogenic effect |
| Other subtypes | NA | NA |
| Brest cancer | AHNAK | AHNAK2 |
| Triple-negative breast cancer | bi-directional regulation | NA |
| Other subtypes | NA | NA |

NA: no applicable corresponding data
